# Impaired expression of DICER and some microRNAs in HBZ expressing cells from acute adult T-cell leukemia patients

**DOI:** 10.18632/oncotarget.7162

**Published:** 2016-02-03

**Authors:** Hélène Gazon, Gildas Belrose, Marie Terol, Jean-Come Meniane, Jean-Michel Mesnard, Raymond Césaire, Jean-Marie Peloponese

**Affiliations:** ^1^ CPBS, CNRS UMR 5236, Université Montpellier 1, Montpellier, France; ^2^ Laboratoire de Virologie-Immunologie JE2503, Centre Hospitalier et Universitaire de Martinique, Fort de France, Martinique; ^3^ Service hématologie clinique, Centre Hospitalier et Universitaire de Martinique, Fort de France, Martinique

**Keywords:** Dicer, miRNAs, AP-1, JunD, HBZ

## Abstract

Global dysregulation of microRNAs (miRNAs), a class of non-coding RNAs that regulate genes expression, is a common feature of human tumors. Profiling of cellular miRNAs on Adult T cell Leukemia (ATL) cells by Yamagishi et al. showed a strong decrease in expression for 96.7% of cellular miRNAs in ATL cells. However, the mechanisms that regulate the expression of miRNAs in ATL cells are still largely unknown. In this study, we compared the expression of 12 miRs previously described for being overexpress by Tax and the expression of several key components of the miRNAs biogenesis pathways in different HBZ expressing cell lines as well as in primary CD4 (+) cells from acute ATL patients. We showed that the expression of miRNAs and Dicer1 were downregulated in cells lines expressing HBZ as well as in fresh CD4 (+) cells from acute ATL patients. Using qRT-PCR, western blotting analysis and Chromatin Immunoprecipitation, we showed that *dicer* transcription was regulated by c-Jun and JunD, two AP-1 transcription factors. We also demonstrated that HBZ affects the expression of Dicer by removing JunD from the proximal promoter. Furthermore, we showed that at therapeutic concentration of 1mM, Valproate (VPA) an HDAC inhibitors often used in cancer treatment, rescue Dicer expression and miRNAs maturation. These results might offer a rationale for clinical studies of new combined therapy in an effort to improve the outcome of patients with acute ATL.

## INTRODUCTION

Adult T-cell leukemia (ATL) is an aggressive human malignancy with a rapid and lethal clinical course [1, 2]. ATL is etiologically associated with human T-lymphotropic virus type I (HTLV-I) and is known to be resistant to standard anticancer therapies [1, 2]. Current findings suggest that HTLV-I induced oncogenesis is due to the action of the two viral proteins Tax and HTLV-1 bZIP factor (HBZ). Tax, the viral transactivator protein regulates not only viral transcription, but interferes with most DNA repair mechanisms, preventing cell cycle arrest and inducing cellular senescence [1, 2]. Tax expression cannot be detected in ~60% of acute ATL cases, which indicates that Tax is only implicated in early steps of cell transformation [1, 2]. Conversely, HBZ which is encoded by the minus strand of the HTLV-1 provirus, promotes proliferation of ATL cells and is consistently expressed in all ATL cells [1–4]. Treatments of patients with ATL include chemotherapy and allogeneic hematopoietic stem-cell transplantation [1, 2]. As far as chemotherapy against ATL is concerned, inhibitors of the topoisomerase II, doxorubicin and etoposide, are frequently used. Monotherapy and polychemotherapy demonstrated a low response rate and poor survival. Low improvement has been reached with zidovudine and interferon [5]. Thus far, none of the drugs used seems to alter the fatal outcome of the disease; hence, new therapeutic approaches are needed.

Recently, much interest has developed in elucidating the cross-talk between tumor development and HTLV-1 infection as it relates to the innate host response and in particular the microRNA network. MicroRNAs (miRNAs) are important post-transcriptional regulators of gene expression [6, 7]. MiRNAs are transcribed by RNA polymerase II as long primary transcripts called pri-miRNA. These long pri-miRNA precursors are processed by the RNase III enzyme Drosha and its cofactor DiGeorge syndrome critical region gene 8 (DGCR8) into hairpin precursors, the pre-miRNA [6, 7]. Then Dicer and its cofactor TRBP cleave the pre-miRNA hairpin separating the loop from the double-stranded stem forming a miRNA duplex [6, 7]. Dicer and the miRNA duplex form a ternary complex with Argonaute (Ago) proteins [6, 7]. The duplex is unwound, giving rise to the active single-stranded miRNA in the RNA-induced silencing complex (RISC) that targets mRNAs for degradation or translational inhibition [6, 7].

Deregulated miRNA expression has been demonstrated in a variety of human cancer types, including chronic lymphocytic leukemia, lung cancer, colorectal neoplasia, and pancreatic endocrine [8, 9]. Furthermore, downregulation of miRNAs and its implication in chemoresistance was also reported in ovarian cancer [10], breast cancer [11] and hepatocarcinoma [12]. We asked whether there is a correlation between the described downregulation of miRNAs in fresh ATL cells by Yamagishi et al. [13], and the observed chemoresistance of ATL cells [14] and we aimed to determine the underlying mechanism.

Here we report that HBZ downregulates Dicer expression in ATL cells. This HBZ-mediated reduction of Dicer led to the decrease in the abundance of numerous miRNAs without parallel declines of the corresponding pre-miRNAs. Through its influence on miRNA biosynthesis, Dicer influences cell-cycle progression, senescence and tumorigenesis [15–17]. Despite its important roles in cellular homeostasis, the mechanisms that control Dicer expression are not well characterized. Here, we show that Dicer expression is regulated by the AP-1 transcription factor JunD in T lymphocytes. We demonstrate that HBZ downregulates Dicer expression by removing JunD from the AP-1 binding site located into *dicer* proximal promoter. Valproate acid (VPA), an anti-seizure agent acting as a histone deacetylase inhibitor (HDACi) at therapeutic concentrations [18], has emerged as a promising anti-neoplastic agent [19]. Indeed through hyperacetylation of histone and subsequent relaxation of chromatin, VPA may enhance the cytotoxicity of drugs targeting DNA [19]. In this study, we show that, at 1mM (i.e. concentration reached in the serum of patients treated for epilepsy), VPA rescues *dicer* expression and miRNAs maturation in ATL cells. Our findings suggest that VPA can be a potent agent to be introduced in clinical assays for treatment of ATL.

## RESULTS

### MiRNAs levels are reduced in HTLV-1-infected cells with high HBZ expression

**Figure 1:**
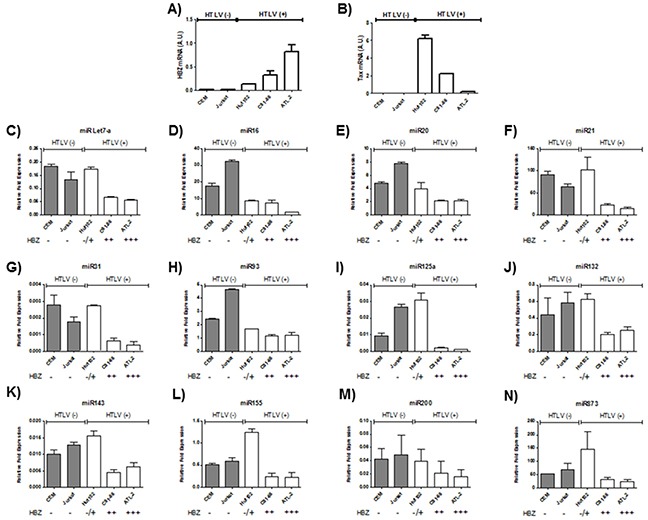
Decreased miRNA levels in HTLV-1 infected cells lines **A-B.** Relative expression of *tax* and *hbz* was measured by quantitative RT-PCR and normalized to HPRT RNA levels in the two controls T-cell lines CEM and Jurkat (grey bars) and the three HTLV-1 infected T-cells lines Hut-102, C81-66 and ATL-2 (white bars). **C-N.** The levels of the indicated miRNAs were measured using qRT-PCR and normalized to U6 snRNA levels in the two controls T-cell lines CEM and Jurkat (grey bars) and the three HTLV-1 infected T-cells lines Hut-102, C81-66 and ATL-2 (white bars). Data are the means ± S.D. from three independent experiments.

**Figure 2:**
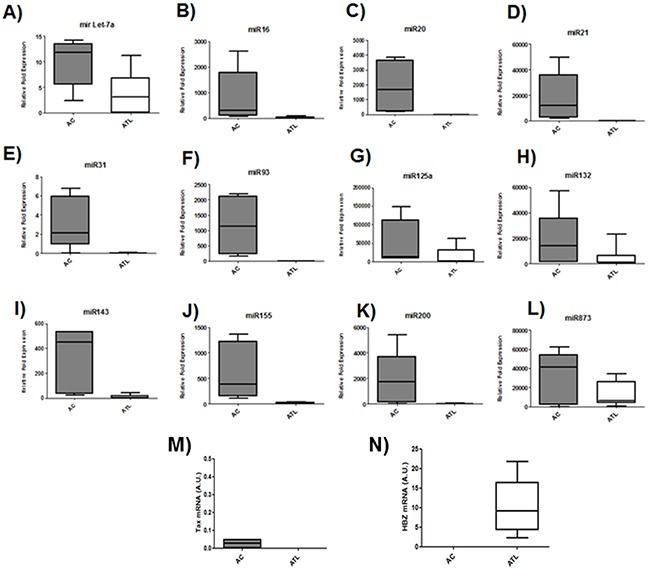
Decreased miRNA levels in ATL patients **A-L.** The levels of the indicated miRNAs were measured using qRT-PCR and normalized to U6 snRNA levels in CD8+-cell–depleted PBMCs from HTLV-1 asymptomatic carriers (AC) and patients with acute ATL (ATL). **M-N.** Relative expression of *tax* and *hbz* was measured by quantitative RT-PCR and normalized to HPRT RNA levels. in CD8+-cell–depleted PBMCs from HTLV-1 asymptomatic carriers (AC) and patients with acute ATL (ATL).

**Figure 3:**
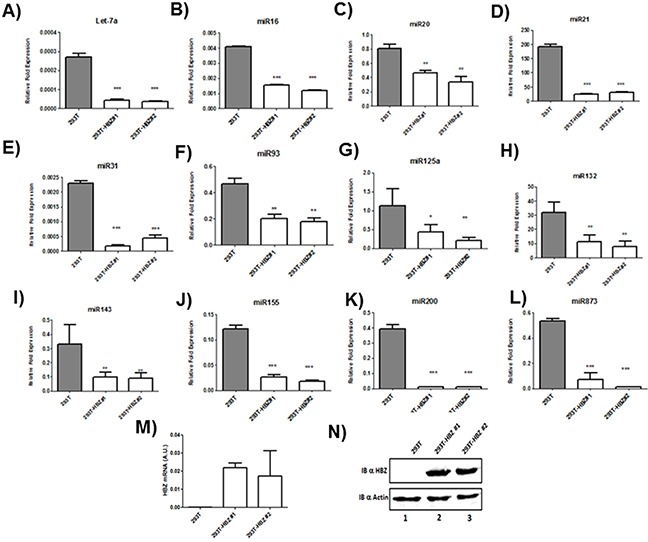
Decreased miRNA levels in 293T cells lines stably expressing HBZ **A–L.** The levels of the indicated miRNAs were measured using qRT-PCR and normalized to U6 snRNA levels in the control 293T cell lines (grey bars) and the two 293T-HBZ cell lines (white bars). Data are the means ± S.D. from three independent experiments (**P<0.01, and ***P<0.001). **M.** Relative expression of *hbz* was measured by quantitative RT-PCR and normalized to HPRT RNA levels. **N.** the level of HBZ in two 293T cells stably expressing HBZ (293T-HBZ#1 et 293T-HBZ#2) (lanes 2-3) were assessed by western blot using an HBZ monoclonal antibody and actin was used as loading control.

Microarray analysis of HTLV-1 infected T-cells lines identified several miRNAs that were significantly up regulated by Tax expression [20, 21]. Among those upregulated miRNAs by Tax, we focused on miRNAs known to play a key role in oncogenesis and chemoresistance such as miRlet7-a, miR16, miR20, miR 21, miR31, miR93, miR125a, miR132, miR143, miR155,miR200 and miR873 [22, 23]. In order to assess the effect of HBZ on miRNA expression, we compared the abundance of miRlet7-a, 16, 20, 21, 31, 93, 125a, 132, 143, 155, 200 and 873 in two uninfected T-cell lines (CEM and Jurkat), one HTLV-1 T-cell line with low HBZ-expression (Hut-102), and two HTLV-1 T-cell lines with high HBZ-expression (C81-66 and ATL-2) (Figure 1) and in HTLV-1 infected cells from asymptomatic carries (AC) and from ATL patients (ATL) (Figure 2). The expression of let-7a, miR16, 20, 21, 31, 93, 125a, 132, 143, 155, 200 and 873 between HBZ expressing T cells and uninfected T-cells was compared by using real-time PCR. We observed that in ATL cells as well as in HTLV-infected-cells lines expressing significant level of HBZ (C81-66 and ATL-2), the miRNAs tested were less abundant than in the high Tax-expressing (Hut102) and uninfected T-cell lines (CEM, Jurkat) (Figures 1–2). To confirm a specific effect of HBZ on miRNAs abundance, we next compared the level of miRNAs expression in 293T vs. 293T stably expressing HBZ, 293T-HBZ (Figures 3 A–L). Indeed, we observed that miRNAs tested were less abundant in HBZ expressing cells than in control 293T cells (Figure 3). These findings suggest that expression of HBZ is associated with decrease of miRNAs abundance previously observed in fresh ATL cells by Yamagishi et al. [13].

### HBZ inhibits miRNA maturation by reducing Dicer1 expression

**Figure 4:**
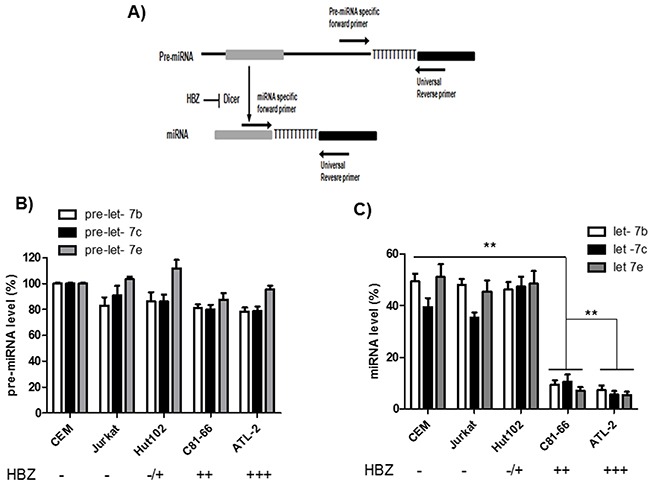
Impaired miRNA maturation in HTLV-1 infected cells lines **A.** Schematic representation of the experimental design to detect pre-miRNAs and mature miRNAs. MicroRNA genes are transcribed as large primary transcripts that are processed by a protein complex containing Drosha, to form an approximately 70 nucleotide hairpin precursor microRNA (pre-microRNA). This precursor is subsequently transported to the cytoplasm where it is processed by DICER, to form a mature microRNA of approximately 22 nucleotides. Pre-miRNAs were detected using forward primers that specifically hybridized with the pre-miRNA (but not the mature miRNA). In both case an reverse universal primer was used fo qPCR amplification. **B-C.** The levels of Let-7 pre-miRNAs (B) and mature miRNAs (C) were measured using qRT-PCR and normalized to U6 in two controls T-cell lines (CEM and Jurkat) and three HTLV-1 infected T-cells lines (Hut-102, C81-66 and ATL-2). Data are the means ± S.D. from three independent experiments (**p<0.01).

Several mechanisms may lead to altered miRNA expression, one of which is a defect of miRNA biogenesis. We examined the canonical miRNA maturation pathway in HTLV-1-infected T cells lines. To this end, we compared the levels of pre-miRNA and mature miRNA for three members of the let-7 family (Figure 4). Briefly, after reverse transcription, mature miRNAs were detected by using a forward primer that hybridized with the miRNAs, while precursor miRNAs (pre-miRNAs) were detected using forward primers specifically designed to hybridize within the pre-miRNA but not the mature miRNA. In both cases, a reverse universal primer was used for qPCR amplification. We observed that the levels of pre-let-7b, pre-let-7c and pre-let-7e were similar in all three HTLV-1 infected cell lines relative to control cell lines. In contrast, in C81-66 and ATL-2 cells, mature miRNAs were less abundant than those measured in CEM, Jurkat and Hut-102 cells (Figure 4). Collectively, these results indicate that T-cell expression of HBZ is associated with impaired miRNA maturation.

**Figure 5:**
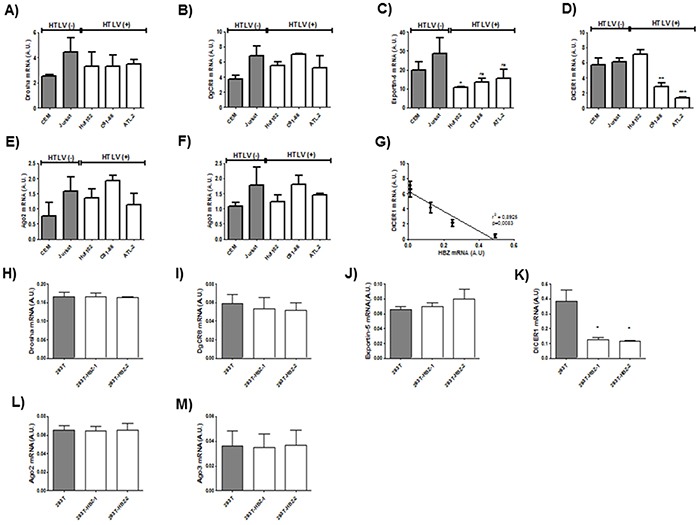
Decreased expression of Dicer in HTLV-1 infected cells lines and in 293T cells lines stably expressing HBZ **A-F.** Relative expression of six genes related to miRNA biogenesis (*drosha, DgCR8, exportin-5, dicer, ago2 and ago3*), *tax*and *hbz* was measured by quantitative RT-PCR and normalized to HPRT RNA levels in the two controls T-cell lines CEM and Jurkat (grey bars) and the three HTLV-1 infected T-cells lines Hut-102, C81-66 and ATL-2 (white bars). Significance difference in relative expression is indicated by asterisk (**p<0.01; ***p<0.001). **G.** Significant correlation between Dicer decreased expression and HBZ relative expression level was shown in T-cells lines. Pearson's correlation coefficient was 0.8925 (*p*<0.01). Data are the means ± S.D. from three independent experiments (** *p*<0.01). **H-M** Relative expression of 6 genes related to miRNA biogenesis (*drosha, DgCR8, exportin-5, dicer, ago2 and ago3*) and *hbz* was measured by quantitative RT-PCR in in the control 293T cell lines (grey bars) and two 293T-HBZ cell lines (white bars). Significance difference in relative expression is indicated by asterisk (**p<0.01; ***p<0.001).

**Figure 6:**
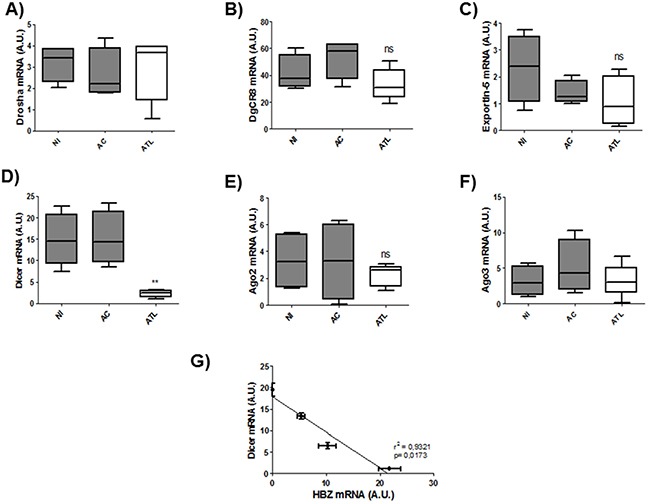
Decreased expression of Dicer in fresh ATL cells **A-F.** Relative expression of six genes related to miRNA biogenesis (*drosha, DgCR8, exportin-5, dicer, ago2 and ago3*) was measured by quantitative RT-PCR and normalized to HPRT RNA levels in CD8+-cell–depleted PBMCs from HTLV-1 asymptomatic carriers (AC) and patients with acute ATL (ATL). Significance difference in relative expression is indicated by asterisk (**p<0.01; ***p<0.001). **G.** Significant correlation between Dicer decreased expression and HBZ relative expression level was shown in ex vivo cultured cells from patients with acute ATL. Data showed HBZ and Dicer expression over 5 days of ex vivo culture (d0,d1,d3 and d5). Pearson's correlation coefficient was 0.9321 (*p*<0.01).

Next to confirm the defect in miRNA maturation, we used quantitative RT-PCR to measure expression of six important components of the miRNA maturation machinery (*Drosha, Dgcr8, Exportin-5, DICER1, Ago 2 and Ago3*) as well as expression of *Tax* and *HBZ* in HTLV-1 infected T-cell lines (Figure 5 A–F) and in 293T stably expressing HBZ (Figure 5 G–L) and in HTLV-1 infected cells from asymptomatic carries (AC) and from ATL patients (ATL) (Figure 6). Interestingly, only *dicer* expression was downregulated ([*p*<0,01], unpaired *t*-test) in HBZ expressing T-cells (Figure 5D and 5J) and in ATL cells (Figure 6D). We then examined correlation according to the relative expression ratio of Tax and HBZ (Figure 1A and 1B). We found that reduced *dicer* expression was correlated with increased expression of HBZ in HTLV-1 infected cells (Figure 5G) (Spearman's rank correlation coefficient r^2^= 0,8925; *p* = 0.0083) and in ATL cells (Figure 6G) (Spearman's rank correlation coefficient r^2^= 0,9321 ; *p* = 0.0173). Taken together, our results suggest that impaired miRNA maturation occurs through a reduction in *dicer* expression in cells that express HBZ.

### HBZ represses miR function *in vivo*

**Figure 7:**
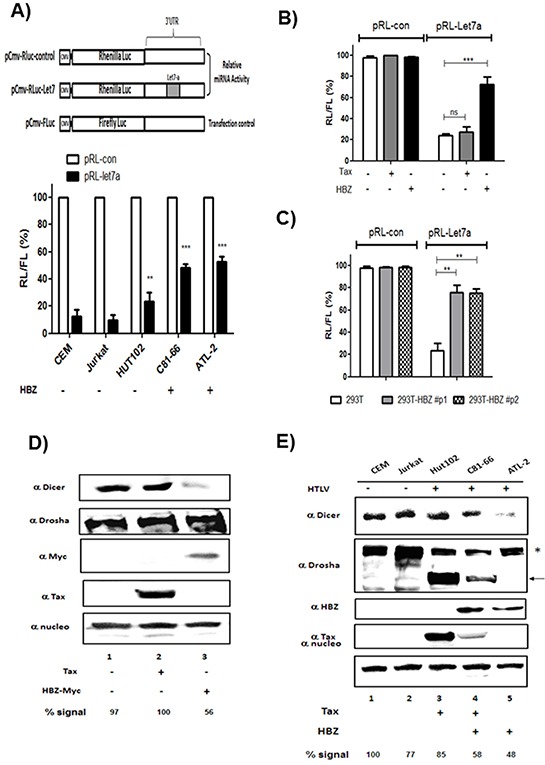
HBZ but not Tax decreases Dicer Expression **A.** Decreased RNA silencing in HTLV-1 infected cells expressing HBZ. HTLV-1 infected cells lines and control cell lines were transfected with a Renilla luciferase (RL)-miR-Let7 reporter plasmid. Activities of Renilla luciferase (RL)-miR-Let7 reporter in each cell line are expressed in relation to activities of RL-control reporter (set as 100%). Values are means ± S.D. of four transfections (**p<0.01; ***p<0.001). **B-C.** HBZ but not Tax decreases the efficiency of RNA interference mediated by the endogenous microRNA miR-let7 in 293T expressing transiently (B) or stably HBZ (C). Activities of Renilla luciferase (RL)-miR-Let7 reporter in each cell line are expressed in relation to activities of RL-control reporter (set as 100%). Values are means ± S.D. of four transfections (**p<0.01; ***p<0.001). **D.** 293T cells were transfected with a control plasmid (pcDNA) (lane 1), Tax plasmid (lane 2) or HBZ-Myc plasmid (lane 3). Forty-eight hours after transfection, Dicer, Drosha, HBZ, Tax and nucleolin expression were assessed by western blotting analysis. **E.** Dicer, Drosha, HBZ, Tax and nucleolin expression were assessed from lysates of two controls T-cell lines CEM and Jurkat (lanes 1 and 2) and HTLV-1 infected T-cells lines Hut-102, C81-66 and ATL-2 (lanes 3-5) by western blot. Dicer signals were quantified by densitometry and represented as a percentage of Dicer levels in the control group.

To assess the ability of HBZ to reverse the inhibitory effects of endogenous miRNAs, we used a miRNA-mediated mRNA-reporter-cleavage assay [24]. Cells were transfected with constructs expressing either control Renilla luciferase (RL) reporter mRNA or Renilla luciferase mRNA harboring a miR-Let7 complementary sequence in the 3′ untranslated region (Figure 7A). We performed assays using HTLV-1- infected T cells lines (Figure 7A) and 293T cells transiently (Figure 7B) or stably (Figure 7C) expressing HBZ. In control cells, insertion of the miR-Let7 site repressed RL expression by ~80%. However, in cells expressing HBZ, the miRNA-mediated inhibition was about three to four fold less pronounced (Figure 7A, 7B and 7C), indicating that HBZ expression impairs miRNA function.

We next examined the effects of HBZ on the expression and stability of Dicer protein. 293T cells were transfected with expression vectors for Tax, HBZ or an empty vector control, and Dicer protein levels were analyzed by Western blot (Figure 7D). While Tax had no effect on the level of Dicer, HBZ reduced the steady-state levels of Dicer protein to 50% (Figure 7D, lane 1 vs. lane 3). In a complementary experiment, we analyzed expression of Dicer and Drosha in HTLV-1 infected cells (Hut-102, C81-66 and ATL-2) and uninfected T cells (CEM and Jurkat) (Figure 7E, lanes 1–2 and lanes 3–5 respectively). We found that the levels of Dicer were decreased by 42% in C81-66 cells and 52% in ATL-2 cells, when compared to CEM cells (normalized to nucleoline). Interstingly while expression level of isoform 1 of Drosha (*) was decreased, a smaller form of Drosha (arrow) was observed in T-cell lines expressing Tax (Hut102 and C81-66) (Figure 7E lanes 3-4). These data show that HBZ expression downregulates Dicer protein.

### HBZ decreases JunD-mediated transcription from the *Dicer* promoter

**Figure 8:**
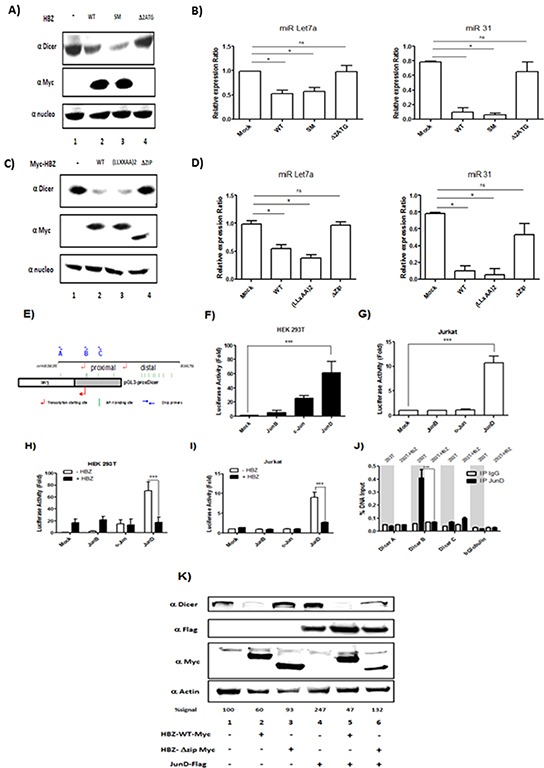
HBZ exerts a negative effect on the transcriptional activation of the Dicer proximal promoter through JunD **A-D.** The leucine zipper of HBZ protein is require to decrease Dicer Expression level.293T were transfected with a control plasmid (pcDNA) (lane 1), HBZ-Myc plasmid (lane 2) and plasmids expressing a functional mutant of HBZ (lanes 3 and 4). Forty-eight hours after transfection, Dicer, HBZ and nucleolin expression were assessed by western blotting analysis (A-C) and miR Let7a and MiR 31 expression were measured by qRT-PCR and normalized to U6. **E.** Schematic representation of the Dicer promoter sequences and the pGL3-prox Dicer Luc construct. The binding sites for AP-1 transcription factors found in Dicer promoter are indicated as green vertical lines. The arrows indicate the locations of primers used to amplify sequences of the proximal promoter. **F-G.** AP-1-mediated activation of Dicer proximal promoter. 293T cells (F) and Jurkat cells (G) were transiently transfected with the pGL3-prox Dicer-Luc reporter plasmid along with indicated expression vectors for the Jun Family. Forty-eight hours later, luciferase activity was analyzed and normalized to β-Galactosidase activity. Data (mean and standard deviation) are representative of three independent experiments. **H-I.** HBZ specifically inhibit JunD-mediated activation of Dicer proximal promoter. 293T cells (H) and Jurkat cells (I) were transiently transfected with the pGL3-prox Dicer-Luc reporter plasmid and with the vector encoding for JunB, c-Jun, JunD and/or HBZ. Forty-eight hours later, luciferase activity was analyzed and normalized to β-Galactosidase activity. Data (mean and standard deviation) are representative of three independent experiments. **J.** HBZ inhibits Dicer expression by removing JunD from endogenous Dicer promoter. 293T controls (293T) and 293T stably overexpressing HBZ (293T-HBZ) were subjected to chromatin immunoprecipitation using anti-JunD antibody or control pre-immune rabbit serum. Purified DNA was analyzed by quantitative RT-PCR using primers spanning the Dicer proximal promoter (primers A, B and C) or the βglobulin promoter (as control). DNA recovered from chromatin samples before immunoprecipitation, which corresponds to 2% of that used for immunoprecipitation, was also amplified as input. Data (mean and standard deviation) are representative of two independent experiments done in triplicates. **K.** Overexpression of JunD increase Dicer level. a control plasmid (pcDNA) (lane 1), HBZ-Myc plasmid (lanes 2 & 5) and plasmids expressing a functional mutant of HBZ (lanes 3 &6) and JunD-Flag (lanes 4-6). Forty-eight hours after transfection, Dicer, HBZ, JunD and actin expression were assessed by western blotting analysis. Dicer signals were quantified by densitometry and represented as a percentage of Dicer levels in the control group.

HBZ has been well documented to function as a transcription factor that forms complexes with the highly related cellular coactivators CBP and p300, and with certain cellular bZIP transcription factors [25, 26]. We sought to determine which sets of interactions may facilitate downregulation of Dicer. We probed HBZ function using HBZ-SM (silent mutations in mRNA), HBZ-Δ2ATG (only produces HBZ mRNA), HBZ-(LXXAA)_2_ (contains mutations that abrogate p300/CBP-binding), and HBZ-ΔZip (lacks the leucine zipper required for interactions with bZIP transcription factors) [25, 26]. We established that wild-type HBZ, HBZ-(LXXAA)_2_ (Figure 8A, lanes 2, 3) and HBZ-SM (Figure 8C, lane 3) decreased expression of Dicer. In contrast, HBZ-ΔZip (Figure 8A lane 4) and HBZ-Δ2ATG (Figure 8C, lane 4) did not affect the level of Dicer. To confirm a specific effect of HBZ on miRNAs maturation, we also compared the level of miRNAs expression in 293T expressing HBZ vs. functional mutant of HBZ (Figure 8B & 8D). Indeed, we observed that in cells transfected with HBZ WT, HBZ-(LXXAA)2 (Figure 8B, lanes 2 & 3) and HBZ-SM (Figure 8D, lane 3) miR Let7a and miR31 were less abundant than in control 293T cells (Figure 8B and 8D, lane 1). Together these results suggest that HBZ inhibits Dicer expression through a protein-protein interaction with bZIP transcription factors.

To elucidate the mechanism by which HBZ deregulates the expression of Dicer, we used *in silico* prediction model and identified thirteen AP-1 binding site sequences in the promoter of Dicer (Figure 8E). Among these targets, we focused on the five AP-1 binding sites located in the proximal promoter. Using a luciferase reporter assay [27], we compared the ability of each member of the Jun family to activate transcription of the promoter in 293T cells (Figure 8F) and in Jurkat cells (Figure 8G). While both c-Jun and JunD significantly activated transcription from the *dicer* promoter in 293T cells (Figure 8F, lanes 3, 4), only JunD increased transcription from the promoter in Jurkat cells (Figure 8G, lane 4). We next assessed whether HBZ impairs JunD-mediated trans-activating of the *dicer* proximal promoter. We found that, in both 293T (Figure 8H, lanes 2, 4) and Jurkat cells (Figure 8I, lanes 2, 4), co-expression of HBZ with JunD decreased luciferase activity by three fold compared to expression of JunD alone (Figures 8H and 8I respectively). To investigate whether HBZ affects the binding of JunD to the endogenous *dicer* promoter, we performed ChIP assays in 293T cells stably expressing HBZ (293T-HBZ) (Figure 8J). Interestingly, JunD was found to be associated with the proximal promoter sequence closest to the transcription start site in control cells (293T) (Figure 5J-Dicer B). We observed a decrease of JunD binding in 293T-HBZ cells, suggesting that in presence of HBZ, JunD binding to the *dicer* promoter is inhibited. To confirm a specific effect of JunD on Dicer expression, 293T were co-transfected with JunD (Figure 8K lanes 4-6) and either HBZ WT (Figure 8K lanes 2 & 5) or an HBZ that lost the ability to interact with JunD (HBZ ΔZip) (Figure 8K lanes 3 & 6). We found that the level of Dicer protein was increased up to 147% in presence of JunD (Figure 8K). Taken together, our results strongly suggest that in T-cells, expression of Dicer is controlled by JunD.

### VPA treatment restore dicer expression and miRNA maturation in HTLV-1 infected cells

**Figure 9:**
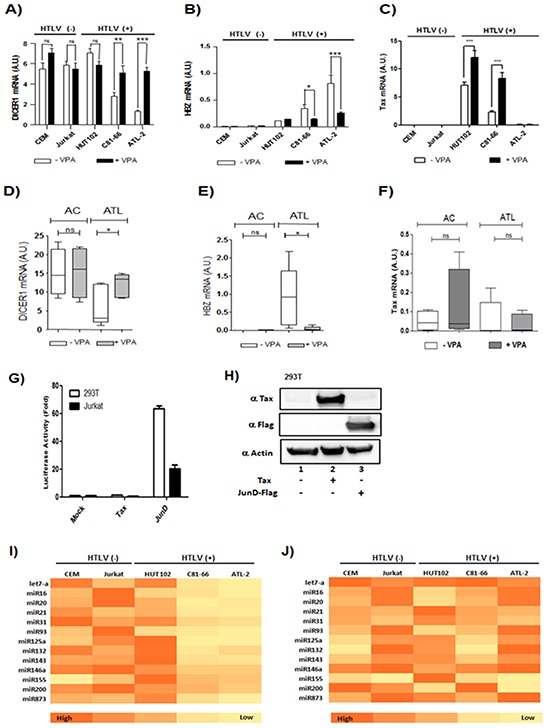
Valproate treatment of CD4+ T cells isolated from ATL patients restore Dicer expression **A-C.** Two controls T-cell lines (CEM; Jurkat) and three HTLV-1 infected T-cells lines (Hut-102; C81-66 and ATL-2) were treated with (black histogram) or without VPA (white histogram). Relative expression of Dicer, Tax and HBZ was measured by quantitative RT-PCR. Significant differences in expression are indicated by asterisk (**p<0.01; ***p<0.001). **D-F.** Relative expression of Dicer, Tax as well as HBZ was measured by quantitative RT-PCR in CD8+-cell–depleted PBMCs from HTLV-1 asymptomatic carriers (AC) and patients with acute ATL (ATL) treated (grey boxes) or not (white boxes) with VPA. Significance difference is indicated by asterisk (*p< 0.05; **p<0.01). **G-H.** AP-1-mediated activation of Dicer proximal promoter. 293T cells and Jurkat cells (G) were transiently transfected with the pGL3-prox Dicer-Luc reporter plasmid along with indicated expression vectors for Tax or JunD. Forty-eight hours later, luciferase activity was analyzed and normalized to β-Galactosidase activity. Data (mean and standard deviation) are representative of three independent experiments. (H) Forty-eight hours after transfection, Tax, JunD and actin expression were assessed by western blotting analysis. **I-J.** Summary heat map of differentially expressed miRNAs. Two controls T-cell lines CEM and Jurkat and the three HTLV-1 infected T-cells lines Hut-102, C81-66 and ATL-2 were treated with or without VPA. Heat map summarizing the patterns of expression for 13 miRNAs that were differentially expressed in HTLV-1 infected cell lines subtypes without VPA (I) and with VPA treatment (J).

We next tested whether extinction of HBZ expression could restore *dicer* expression. However, since Dicer is a key component of the RNAi pathway [6, 24], we choose not to use siRNA to knockdown HBZ expression. As we previously described, treatment with Valproic acid (VPA), a histone deacetylase inhibitor, impaired HBZ expression in HTLV infected cells [28]. We treated HTLV-1 infected cells lines and CD8+ depleted PBMCs from AC or from ATL with 1mM VPA for 48 hours. Using qRT-PCR, we measured the expression levels of *dicer, tax* and *hbz* in HTLV-1 infected cells lines (Figure 9 A–C) and in CD8+ depleted PBMCs from ACs and from acute ATL patients (Figure 9 D–F). We found that VPA treatment resulted in the restoration of *dicer* concomitant with decreased levels of *hbz*. This inverse correlation was observed in both cell lines (Figure 9B) and patient samples (Figure 9E). We found no significant correlation between an increase of *tax* expression and restoration of *dicer* expression. Indeed while VPA treatment increase *tax* expression in HTLV-infected cell lines (Figure 9C), no similar effect was observed in ATL patient samples (Figure 9F). Next, using a luciferase reporter assay, we compared the ability of Tax and JunD to activate transcription of the promoter of dicer in 293T cells and Jurkat (Figure 9G–9H). While JunD significantly activated transcription from the *dicer* promoter (Figure 9G), Tax was unable to activate transcription from the promoter in Jurkat cells (Figure 9G). To further confirm these results, we used qRT-PCR to assess the level of 13 miRNAs before (Figure 9I) and after VPA treatment (Figure 9J). As illustrated in the heat map representation of relative miRNA levels, VPA rescues the expression of hbz-downregulated miRNAs. Collectively, these results indicate that HBZ downregulates dicer gene expression, in turn decreasing the levels of mature miRNAs in the ATL cells.

**Figure 10:**
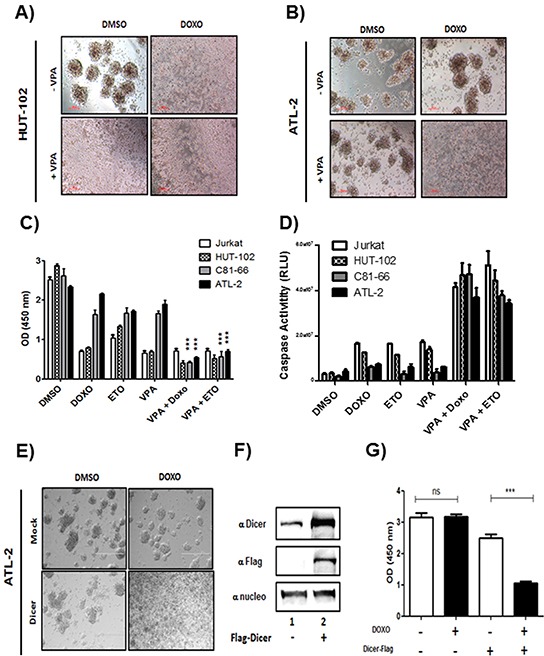
doxorubicin and etoposide-induced cell death in ATL cells lines co-treated with Valproate control T-cell line Jurkat and the three HTLV-1 infected T-cells lines Hut-102, C81-66 and ATL-2 were treated with doxorubicin (1μM), etoposide (100μM) or Valproic acid (1mM) for 24 h **A-B.** ATL-2 cells are resistant to doxorubicin treatment. Morphology of HTLV-1 infected cells Hut-102 (A) and ATL-2 (B) before and after VPA and/or doxorubicin treatment. Healthy cells are piling up to form foci while lost of foci is observed with apoptotic cells **C.** Cell viability for each cell lines was measured with CCK8 Dojindo ® kit according to manufacturer instruction and confirmed with Trypan Blue exclusion assay. **D.** Apoptosis was measured with Caspase 3-7 GLO kit from Promega according to manufacturer instruction. **E-F.** ATL2 cells overexpressing Dicer are sensitive to Doxorubicin. ATL-2 cells were transfected using lipofectamine 3000 reagent with a plasmid expressing Dicer tagged Flag. 48h after transfection, cells were treated with G418 to sort transfected cells only. Morphology of ATL-2 transfected with DicerFlag (E) before and after doxorubicin treatment. Healthy cells are piling up to form foci while lost of foci is observed with apoptotic cells (F) Dicer and nucleolin expression were assessed by western blotting analysis. **G.** Cell viability was measured with CCK8 Dojindo ® kit according to manufacturer instruction and confirmed with Trypan Blue exclusion assay.

Repression of Dicer and subsequent downregulation of miRNAs is associated with invasive phenotype and chemoresistance in ovarian cancer, glioblastoma, human cutaneous melanoma or hepatocellular carcinoma [10, 15, 22, 23, 29]. To verify the effect of Dicer knockdown on sensitivity of ATL cells to doxorubicin (Doxo) and etoposide (Eto), two drugs frequently used to treat ATL, cells viability and apoptosis were assessed by CCK-8 assays and Caspase 3/7 Glo assays following treatment with Doxo or Eto in absence or presence of VPA (Figure 10). While Hut-102 (low HBZ), showed apoptotic figures when treated with 1μM doxorubicin alone or with 1mM VPA (Figure 10A), ATL-2 (high HBZ), did not show apoptotic figures when treated with Doxo or with VPA alone (Figure 10B). However, ATL-2 cells showed apoptotic figures when co treated with Doxo and VPA (Figure 10B). A similar pattern was also observed when Hut-102 and ATL-2 were treated with 100 μM etoposide (data not shown). Co-treatment with VPA/ Doxo or with VPA/Eto decrease cell viability of C81-66 and ATL-2 by ~80% when compared to treatment with Doxo or Eto alone (Figure 10C, lanes 2, 3 vs. lanes 5, 6). Since our data established that viability of C81-66 and ATL-2 cells was reduced by the co-treatment, we investigated whether VPA sensitization of C81-66 and ATL-2 cells to Doxo and/or Eto resulted from apoptosis. We measured apoptosis by caspase 3 and caspase 7 activation. As reported in Figure 10D, Doxo alone or Eto alone had no effect on caspase 3 and caspase 7 activation in C81-66 or ATL-2 (Figure 10D, lanes 2,3, grey and black histogram), while Doxo and Eto significantly activated caspase in both Jurkat and HUT-102 cells (Figure 10D, lanes 2,3, white and hatched histogram). In contrast, exposure to VPA resulted in a significant increase in activation of caspase 3/7 in both C81-66 and ATL-2 (Figure 10D, lanes 5,6, grey and black histogram). To further confirm these results, we transfected ATL2 with expression vector for Dicer-Flag. Dicer expression and cells viability were assessed by western blotting and CCK-8 assays following treatment with Doxo (Figure 10 E–F). Mock-transfected ATL-2 did not show apoptotic figures when treated with Doxo. However, Dicer-transfected ATL-2 cells showed apoptotic figures when treated with Doxo (Figure 10). Treatment with Doxo decrease cell viability of Dicer-transfected ATL2 by ~60% when compared to mock-transfected cells (Figure 10G). These results establish that VPA treatment sensitizes ATL cells to chemotherapeutic agents by restoring Dicer expression and miRNA maturation.

## DISCUSSION

The mechanisms by which HTLV-1 can transform infected cells are diverse and our understanding of them is constantly evolving. In addition to viral proteins, miRNAs have also been implicated as central players in the development of cancers [8, 23, 30]. Alterations of the microRNA machinery components are thought to elucidate abnormal miRNA profiles in various cancers. Different groups have previously reported down and up-regulation of miRNAs in Tax-expressing cells such as miR93, 144, 146a, 155, 451 [13, 31, 32] however, it is not clear whether the observed up- or downregulation of miRNA expression simply reflects malignant weakening of the tumor or is a direct cause for initiation and tumoral progression [13, 20, 33, 34]. Furthermore, Seishi Ogawa and colleagues recently report the results of an integrated genomic and transcriptomic analysis in an impressive cohort of 426 ATL cases [35, 36]. Using whole-genome sequencing data to map the exact integration site of the HTLV-1 viral genome, Kataoka et al. confirmed that most of ATL cases have only a single viral integration site and that the viral genome was often partially deleted or mutated [35, 36]. Their transcriptomic analysis confirmed repression of the sense transcripts, including tax. In contrast, the antisense transcript HBZ was typically not affected and was expressed at high levels [35, 36]. Vernin et al. reported that the viral factor HBZ promotes genetic instability by activating the expression of the miR 17 and 21 in HTLV-1-infected cells [37]. In this study, we compared the expression of 12 miRs previously described for being overexpress by Tax including miR21 and the expression of several key components of the miRNAs biogenesis pathways in different HBZ expressing cell lines as well as in primary CD4 (+) cells from acute ATL patients. Using miRNA-mediated mRNA-reporter-cleavage assay and quantitative RT-PCR, we observed that HBZ impairs the maturation of miRNAs without changing the level of pre-miRNAs in the cell (Figures 1, 2, 3 and 4). Our results are confirming the findings by Yamagishi et al. [13] for a decrease in mature miRNAs in cells expressing HBZ.

Next, we investigated the regulation of miRNA biogenesis machinery in fresh CD8+ depleted PBMCs isolated from patients with acute ATL. By the use of qRT-PCR and western blotting, we measured the expression level of enzymes essential for the maturation of miRNAs such as Drosha, Dicer, DGCR8, Exportin-5 or Ago2 and Ago3. Interestingly, we only observed a decrease of Dicer expression in HTLV-1 infected cells lines expressing HBZ as well as in fresh ATL cells (Figures 5, 6 and 7). At the difference of the results reported by Van Duyne et al. [34], we observed that the level of pre-miRNAs was not impaired in Hut102 cells (i.e. Tax/Rex-expressing cells) and in C81-66 (Tax-expressing cells). While western blot analysis confirmed that the main isoform of Drosha was downregulated by 50% in Tax-expressing cells (Figure 7E, lanes 3-4, star), we observed that in Hut102 and in C81-66, expression of a shorter isoform of Drosha (Figure 7E–7arrow). This would explain why no significant decrease in pre-miRNA levels were observed in Tax-expressing cells.

Although Dicer itself is not a tumor suppressor gene, a decline in the levels of mature miRNAs is a hallmark of cancer [8, 9]. Indeed, the heightened levels of proteins involved in invasion, metastasis and proliferation have been linked to the reduced levels of miRNAs [8, 9]. Some studies showed higher Dicer expression levels in colorectal, ovarian and prostate cancers [38–40]; however, many other reports have documented declines in Dicer abundance in tumors [15, 41, 42]. In non-small-cell lung carcinoma, Dicer levels were lower in areas of invasion and advanced carcinomas, and reduced Dicer mRNA abundance was associated with poor patient survival [43]. Similarly, in breast, liver, ovarian and bladder cancers, Dicer mRNA levels were significantly lower than in non-cancer tissues [15, 41, 44, 45]; a further association was noted between Dicer protein levels and tumor stage, decreased survival, and chemoresistance [15, 22, 29]. Regardless of growing evidence that Dicer mRNA levels differ in tumors, the regulation of its expression is poorly understood. Dicer gene mutations have been found in humans, and alterations of the Dicer gene were detected in some pre-cancerous and invasive lung adenocarcinomas [46]. In our study, we observed that JunD is able to regulate the expression of Dicer (Figures 7 and 8). We confirmed by chromatin immunoprecipitation assay that JunD binds to dicer promoter sequences (Figure 8J). Interestingly, the region which showed the highest degree of amplification after pulldown of endogenous JunD, contains two adjacent AP-1-binding sites (Figure 8E), which may explain the significance of JunD binding to this region. Reduction of Dicer promoter activity upon HBZ expression as shown by luciferase reporter assay and Chip assay (Figures 8E–J) further confirms the JunD-mediated regulation of Dicer transcription in T- cells.

Conventional treatment of the aggressive forms of ATL (acute and lymphoma) with combination of chemotherapy, in particular those designed for treatment of aggressive non Hodgkin lymphomas or acute lymphoblastic leukemia have little impact on long-term survival of HTLV-1 infected patients [47]. This is mainly due to the intrinsic chemoresistance found in ATL [47]. The requirement for novel treatment modalities is critical to combat this disease [47]. Experimental evidences demonstrate that deregulation of miRNAs biogenesis often leads to drug resistance [15, 22, 29]. In this study, we used VPA in combination with two chemotherapeutic agents, doxorubicin and etoposide, to determine if VPA could potentiate their cytotoxicity on ATL cells. The choice of VPA over others HDAC inhibitors for our studies was based on the facts that VPA is an anti-seizure agent with a well-established toxicity, pharmacological profile in adults and that in a previous study, we have demonstrated that VPA treatment have an opposite effect on the kinetic of Tax and HBZ expression [28]. Our experiments show that VPA by diminishing HBZ mRNA restores Dicer expression and miRNAs maturation in both cells lines and ATL cells (Figure 9). VPA enhances the cytotoxicity of doxorubicin and etoposide on two HBZ-expressing cells lines *in vitro* (Figure 10). Our results are similar to those obtains with others studies where VPA has been reported to enhance the efficacy of chemotherapy in EBV-transformed lymphoblastoid cells [48]. To sum up all the facts, expression levels of the most important enzyme of the miRNA machinery, Dicer was deregulated in ATL cells when compared to asymptomatic carriers. VPA, a clinically available HDAC inhibitor, notably increases apoptosis induced by doxorubicin and etoposide in HBZ-expressing cells. We are aware that our *in vitro* results must be further validated with others HDACIs. However, taking into account these limits, these results might offer a rationale for clinical studies for new combined therapies, in an effort to improve the clinical outcome of patients with adult T-cell leukemia.

## MATERIALS AND METHODS

### Subjects

Blood samples from HTLV-1 infected patients and non-infected donors were obtained from the CHU of Fort-de-France in Martinique. Patients suffering from ATL or HTLV-1 AC were recruited according to World Health Organization (WHO) criteria. According to the French Bioethics laws, the collection of samples from ATL, AC has been declared to the French Ministry of Research and the study was reviewed and approved by the CPP Sud-Ouest/Outre-Mer III, as well as the ARH from Martinique. Because the protocol is non-interventional (e.g. blood samples collected for routine health care with no additional samplings or specific procedures for subjects), no informed consent was provided by the patient, as stated by the French Public Health code and therefore the study was conducted anonymously. Clinical collection of samples for research purpose are stored at the Center of Biological Resources of Martinique (CeRBiM). The CeRBiM database has been approved by the CNIL. Table 1 summarizes the patient's characteristics. AC had no neurologic or hematological symptoms.

**Table 1:** Clinicopathological data for the 10 HTLV infected patients

Characteristic		n
Sex	Male	2
	Female	8
Median age, Year (Range)		50 ± 16
Clinical status	Asymptomatic	4
	Chronic ATL	1
	Acute ATL	5

### Cell culture, transfection and treatments

HTLV-I negative CEM (ATCC® CCL-119™), Jurkat (ATCC® TIB-152™) and HTLV-I-positive HUT-102 (ATCC® TIB-162™), C81-66 and ATL-2 human T-cell lines, were propagated in RPMI 1640 with 10% fetal calf serum (FCS) and transfected using previously described Polybrene/DMSO transfection protocol [49]. 293T cells were propagated in DMEM with 10% FCS and transfected according to manufacturer's protocol using Jet Pei (Polyplus Transfection, Illkrich, France). CD8 (+)-deprived PBMCs from HTLV-1 infected patients were isolated and cultured as previously described in Belrose et al. [28]. Briefly, CD8+-depleted PBMC were cultured at 10^6^/ml in RPMI 1640, supplemented with 10% fetal calf serum, glutamine (2 mM/L), penicillin (100 IU/mL), and streptomycin (100 μg/mL) (Eurobio, Paris, France). When appropriate, valproate (2-n-propylpentanoic acid, VPA) (Sigma-Aldrich, Saint-Quentin Fallavier, France) was added to the medium.

### Luciferase assay

Luciferase assays were performed as previously described [50]. Briefly, 293T or Jurkat cells were transfected with a plasmid DNA mixture containing 100 ng of indicated reporter plasmids and 100 ng of either pActin-bgal or pCMV-FLuc. 48h post-transfection, cells were washed with cold PBS and then lysed in 1x passive lysis buffer (Promega, Charbonnieres, France). Luciferase and β-galactosidase assays were both performed in a Centro XS3 LB 960 microplate luminometer (Berthold, Thoiry, France) with respectively the Genofax A, the Genofax B kit (Yelen, Ensue la Redonne, France) and Galacto-Star kit (Life Technologies, Saint-Aubin, France) as described by the manufacturer. Luciferase activities were normalized for transfection efficiency based on either β-galactosidase or Firefly luciferase readings.

### Western blot analysis

Whole-cell lysates were prepared using RIPA buffer [10 mM Tris–HCl (pH 7.4), 150 mM NaCl, 1% NP-40, 1 mM EDTA, 0.1% SDS and 1 mM DTT], separated by electrophoresis on SD-PAGE gels, and transferred onto PVDF membranes (Millipore). Incubations with primary antibodies to detect Tax (NIH reagent), HBZ (Eurogentec), Dicer, Drosha (Bethyl Laboratory), Nucleoline (Santa Cruz Biotech) or Myc (Sigma-Aldrich) were followed by incubations with the appropriate secondary antibodies conjugated with horseradish peroxidase (HRP) (GE Healthcare) and by detection using enhanced luminescence (Roche). Protein bands were quantified following scanning by ImageJ software (http://imagej.nih.gov/ij/) [51].

### RNA analysis

Total RNA was prepared from whole cells using Trizol (Invitrogen) as previously described [28]. Briefly, after reverse transcription (RT) using oligo-dT 12-18 primer (Invitrogen), the abundance of transcripts was assessed by real-time, quantitative qPCR analysis using the SYBR green PCR master mix (Roche Diagnostics) and gene-specific primer sets. Standard curves were generated from each PCR plate for all primer pairs using a serial dilution of an appropriate experimental sample. Samples were amplified in triplicate on each plate. Data were analyzed using LightCycler®480 Software (Roche Diagnostics). To measure the abundance of mature and precursor miRNAs, we used the QuantiMir detection kit (System Biosciences) according to manufacturer's instructions. Briefly, all cellular RNA were polyadenylated using Poly(A) polymerase, whereupon the oligo-dT adaptor was added to the reaction and annealing was allowed to proceed. After RT, mature miRNAs were detected with forward primers that hybridized with the miRNAs. Pre-miRNAs were detected using forward primers that specifically hybridized with the pre-miRNA (but not the mature miRNA). In both cases, a reverse universal primer was used for qPCR amplification and small nuclear RNA U6, was used for normalization. Primer sequences for Drosha, Dicer, DgCr8, Exportin-5, Ago 2 and 3, HBZ, Tax, HPRT-1 (housekeeping gene) as well as sequences for precursor and mature miRNAs are listed in Supplementary Table S1.

### ChIP assay and quantitative real-time (qRT) PCR analysis

Chromatin immunoprecipitation assays (ChIP) were performed as previously described [50]. Briefly, proteins from 293T and 293T-HBZ cells were cross-linked to DNA with 1% formaldehyde in PBS at room temperature. Chromatin was fragmented by sonication using a Bioruptor (Diagenode) to an average length of 200–500 bp. To reduce non-specific background, antibodies were pre-incubated with Dynabeads® Protein G (Invitrogen) according to manufacturer instruction. Antibodies-beads complexes were then added to chromatin samples and incubated overnight at 4°C. Chromatin was eluted from the beads in elution-buffer (100 mM NaHCO3, 1% SDS) at room temperature. DNA was incubated and purified by proteinase K digestion. For preparation of input controls, samples were treated identical to IP samples except that non-specific antibodies were used. qPCR analysis was performed using LightCycler 480 SYBR Green I Master Mix on LightCycler 480 thermocycler (Roche Diagnostics). Primer sequences for Dicer promoter and β–G1obin promoter are listed in Supplementary Table S1. The average Ct-value was determined from triplicate reactions and normalized against non-specific IgG controls with standard curves for each primer pair. The identities of the products obtained were confirmed by melting curve analysis.

### Statistical analysis

(1) Pearson's correlation for two-dimensional hierarchical clustering analysis; (2) two-tailed pared Student's t test or 2-way ANOVA for *in vitro* cell lines and primary cells experiments, including luciferase assay, RT-PCR, ChIP assay, cell growth assay. Data are presented as mean ± SD. Differences were considered significant at **P<0.01, and ***P<0.001.

## SUPPLEMENTARY TABLE


