# Supplementary material for: Impaired expression of DICER and some microRNAs in HBZ expressing cells from acute adult T-cell leukemia patients
Source: Oncotarget. 2016 Feb 3;7(21):30258–75. doi: 10.18632/oncotarget.7162 (PMC5058679; doi:10.18632/oncotarget.7162)
Supplement: Supplementary file 1 [file oncotarget-07-30258-s001.pdf]

Impaired expression of DICER and some microRNAs in HBZ expressing cells from acute adult T-cell leukemia patients

SUPPLEMENTARY TABLE

Supplementary Table S1: qRT-PCR primers set.

Primer	Forward	Reverse
**qRT-PCR**		
**Drosha**	TCTCTGGAAAGGTCCTACAAAAA	CAGGTTCAGGAACAACCGATA
**Dgcr8**	AAAACTTGCGAAGAATAAAGCTG	TCTGTTTAACAAAGTCAGGGATGA
**Exportin-5**	TATATACTCTCCGCCGCACA	GCCTCTTCAGAAAGACGTAGTGT
**Dicer1**	GTGGTTCGTTTTGATTTGCCC	CGTGTTGATTGTGACTCGTGGA
**Ago2**	GACACGAAAATCACCCACCC	AGGACGTGATAGTGCGAAGG
**Ago3**	TGTGGGCTATCGCTTGTTTT	TGTGGGCTATCGCTTGTTTT
**Tax**	CCAACACCATGGCCCACTT	GATGGGGTCCCAGGTGATCT
**HBZ**	ATGGCGGCCTCAGGGCTGT	TGGAGGGCCCCGTCGCAG
**HPRT1**	TGACACTGGCAAAACAATGCA	GGTCCTTTTCACCAGCAAGCT
**CHIP**		
**Dicer A**	TAACACGGGCTGAAATATAGG	CTGTATCCGTTCTAATGGTCTATC
**Dicer B**	ACTAGGACAGGTGTGAGGGAC	TCAGTAGAGACGGGGTTTCAC
**Dicer C**	AAATTAGCTGGGTGTGGTGG	GGTGCTGAAACTGCTTCCTG
**β-Globin**	AGGCTGCTGGTTGTCTACCCTTG	AGCTCACTGAGGCTGGCAAAGGTG

Primer	Forward	Primer	Forward
**miRNAs**	**http://www.mirbase.org/**		
let-7a	CTATACAATCTACTGTCTTTC	93	CAAAGTGCTGTTCGTGCAGGTAG
let-7b	CTATACAACCTACTGCCTTCCC	125a	TCCCTGAGACCCTTTAACCTGTGA
let-7c	TAGAGTTACACCCTGGGAGTTA	132–3p	TAACAGTCTACAGCCATGGTCG
let-7e	CTATACGGCCTCCTAGCTTTCC	143–3p	TGAGATGAAGCACTGTAGCTC
16	**TAGCAGCACGTAAATATTGGCG**	155:	TTAATGCTAATCGTGATAGGGGT
20a	TAAAGTGCTTATAGTGCAGGTAG	200a:	CATCTTACCGGACAGTGCTGGA
21	TAGCTTATCAGACTGATGTTGA	873:	GCAGGAACTTGTGAGTCTCCT
31	AGGCAAGATGCTGGCATAGCT	U6	**CACCACGTTTATACGCCGGTG**
Precursor let7-b	CTATACAACCTACTGCCTTCCCTG		
Precursor let7-c	CAACCTTCTAGCTTTCCTTGGAGC		
Precursor let7-e	TACGGCCTCCTAGCTTTCCCCAGG		
